# Best clinical practice for imaging in cystic fibrosis referral centers in Brazil: a Delphi consensus panel approach

**DOI:** 10.36416/1806-3756/e20250201

**Published:** 2025-07-22

**Authors:** Leonardo A Pinto, Antonio Fernando Ribeiro, Edna Lúcia D’ Souza, Luciana F V Monte, Vinicius Gomes, Sergio San Juan Dertkigil, Isabela Silva Müller

**Affiliations:** 1. Escola de Medicina, Pontifícia Universidade Católica do Rio Grande do Sul -PUCRS - Porto Alegre (RS) Brasil.; 2. Núcleo de Pesquisa, Grupo Brasileiro de Estudos de Fibrose Cística - GBEFC - São Paulo (SP) Brasil.; 3. Departamento de Pediatria, Faculdade de Ciências Médicas, Universidade Estadual de Campinas - UNICAMP - Campinas (SP) Brasil.; 4. Serviço de Pneumologia Pediátrica e Fibrose Cística, Hospital Universitário, Departamento de Pediatria, Universidade Federal da Bahia - HU-UFBA - Salvador (BA) Brasil.; 5. Coordenação de Pneumologia Pediátrica, Centro de Referência em Fibrose Cística, Hospital da Criança de Brasília José Alencar - HCB - Brasília (DF) Brasil.; 6. Serviço de Radiologia, Hospital da Criança de Brasília José de Alencar - HCB - Brasília (DF) Brasil.; 7. Serviço de Radiologia, Hospital de Base do Distrito Federal, Brasília (DF) Brasil.; 8. Serviço de Radiologia, Faculdade de Ciências Médicas, Universidade Estadual de Campinas - UNICAMP - Campinas (SP) Brasil.; 9. Centro Integrado do Tórax e de Diagnóstico Oncológico da Clínica AMO, DASA, Salvador (BA) Brasil.

## INTRODUCTION

Cystic fibrosis (CF) is a genetic disease characterized by morbidity and mortality related to progressive lung disease. Evidence of structural lung disease occurring early in life provides the opportunity to tailor therapies.^1^ Although the progression of lung disease is routinely assessed by pulmonary function tests,^1^ chest imaging may be more sensitive for the detection of structural lung damage.^2^ The use of cross-sectional imaging modalities such as CT and MRI may contribute to the development of patient-tailored therapy.^2^ However, for most clinicians, it is unclear precisely when, and how, to use chest X-ray (CXR), CT, and MRI. Standard procedures concerning lung imaging are still highly heterogeneous across CF centers.^3^ To address this issue, a Delphi panel of experts in Brazil discussed recommendations for the timing and choice of lung imaging techniques in the assessment of stable or exacerbated CF-related lung disease*.*

## METHODS

We used the Delphi method to determine and quantify group consensus.^4^ The working group consisted of pulmonologists, CF clinicians, and radiologists, representing broad experience in CF and image interpretation. The survey was initially organized around key statements facing CF clinicians in the use of imaging, when to obtain them, and how to act upon the results of the testing. The statements were developed on the basis of previous studies.^2^ ^,^ ^3^ The appropriateness of statements was analyzed with a Likert scale ranging from 0 (completely disagree) to 10 (completely agree). In round 1, the participants were provided with the opportunity to add comments in support of their opinions or to suggest alternate wording for clarity. In round 2, expert participants rated the statements from online questionnaire 2-in this round, there is no need to justify the disagreements. Facilitators analyzed the agreement among panelists-for each statement, agreement was considered to have been achieved if at least 80% of panelists rated the statement as “agree” or “completely agree”. In round 3, the results of the Delphi panel are discussed in an online meeting to evaluate the statements in depth, identifying those for which the level of disagreement was greatest and those that were the most relevant for clinical practice.

## RESULTS AND CONCLUSIONS

Twenty-two recommendations are presented below, including recommendations for diagnosis, follow-up, exacerbations, and radiation dose. Chart 1 shows the main recommendation statements that may be used to inform decisions regarding best clinical practices in CF imaging.

**Chart 1:** Main recommendation statements that may be employed to inform decisions and determine best clinical practices in imaging for patients with cystic fibrosis.

Statement: Current best clinical imaging practice at various CF centers is performing CT biennially (i.e., once every 2 years), with a radiation dose determined by adhering to the ALARA principle, the use of which results in a reasonably low risk related to the cumulative dose (a total of nine CTs from 1 to 17 years of age).
Statement: CT provides relevant information possibly capable of modifying disease trajectory, patient management, and follow-up, in uncooperative and cooperative patients.
Statement: Although MRI of the chest can be considered a surrogate marker for disease severity and treatment response during the short-term follow-up of cooperative patients with CF who are symptomatic or in decline, its use in clinical practice is hampered by its higher cost in comparison with CT; the need for state-of-the-art MRI systems; the occasional need for moderate sedation or general anesthesia in uncooperative children; nonuniformity of MRI protocols; and substantial image variability/capability among MRI manufacturers.
Statement: Despite many specialist centers are using chest X-ray as the imaging technique of choice in infants and preschool children, a CT scan has higher sensitivity for detecting early abnormalities in symptomatic and asymptomatic patients with CF, regardless of age.
Statement: Although low-dose CT is feasible in cooperative and uncooperative patients, its limitations should be considered, especially in uncooperative patients.
Statement: During severe pulmonary exacerbation, CXR can be used. However cases that are more severe might require additional imaging examinations. Alternatives to CXR include low-dose CT (preferable) and lung ultrasound, if available.
Statement: The CT radiation dose should be ALARA without affecting the diagnostic quality of the image. Radiology departments should be encouraged to register the dose-length product in the report for each examination to ensure low-/ultra-low-dose CT and control the cumulative dose.
Statement: For quality control, the dose reports of the CT examinations should be provided by all centers, to track and manage the radiation dose received by patients with CF.
CF: cystic fibrosis; ALARA: as low as reasonably achievable; and CXR: chest X-ray.

Statement 1: In infants diagnosed with CF via newborn screening, low-dose CT can be used as a sensitive tool to detect early disease and monitor disease progression in symptomatic and asymptomatic patients alike. In clinical practice, it could estimate individual response to standards of care in order to estimate the appropriateness of the treatment the patient is given. 

Statement 2: Current best clinical imaging practice at various CF centers is performing CT biennially (i.e., once every 2 years), with a radiation dose determined by adhering to the as low as reasonably achievable (ALARA) principle, the use of which results in a reasonably low risk related to the cumulative dose (a total of nine CTs from 1 to 17 years of age).

Statement 3: CT can detect lung disease progression better than can standard pulmonary function testing parameters (e.g., FEV_1_), in cooperative and uncooperative patients, irrespective of disease severity.

Statement 4: CT provides relevant information possibly capable of modifying disease trajectory, patient management, and follow-up, in uncooperative and cooperative patients.

Statement 5: Despite the fact that treatment with a CF transmembrane conductance regulator (CFTR) modulator results in improvements on imaging (reductions in mucous plugging and peribronchial thickening, etc.), no deviation from the usual imaging follow-up scheme should be advised in patients undergoing such treatment.

Statement 6: Although MRI of the chest can be considered a surrogate marker for disease severity and treatment response during the short-term follow-up of cooperative patients with CF who are symptomatic or in decline, its use in clinical practice is hampered by its higher cost in comparison with CT; the need for state-of-the-art MRI systems; the occasional need for moderate sedation or general anesthesia in uncooperative children; nonuniformity of MRI protocols; and substantial image variability/capability among MRI manufacturers.

Statement 7: Routine use of CT for short-term follow-up during pulmonary exacerbation is not recommended, because of the risk of a high cumulative radiation dose. Clinicians should consider the risk/benefit ratio related to the radiation dose when prescribing CT during pulmonary exacerbation, which should be performed at a low dose or ultra-low dose.

Statement 8: Despite the fact that many specialist centers are using CXR as the imaging technique of choice in infants and preschool children, a CT scan has higher sensitivity for detecting early abnormalities in symptomatic and asymptomatic patients with CF, regardless of age.

Statement 9: CT can detect acute structural lung abnormalities (e.g., increase of bronchial wall thickening and mucus plugging) during and after pulmonary exacerbation, in cooperative and uncooperative patients.

Statement 10: Although low-dose CT is feasible in cooperative and uncooperative patients, its limitations should be considered, especially in uncooperative patients.

Statement 11: During severe pulmonary exacerbation, CXR can be used. However cases that are more severe might require additional imaging examinations. Alternatives to CXR include low-dose CT (preferable) and lung ultrasound, if available.

Statement 12: In chest MRI, the risk related to moderate sedation or anesthesia needs to be considered in uncooperative patients.

Statement 13: The CT radiation dose should be ALARA without affecting the diagnostic quality of the image. Radiology departments should be encouraged to register the dose-length product in the report for each examination to ensure low-/ultra-low-dose CT and control the cumulative dose.

Statement 14: The cancer risk related to the cumulative radiation dose from CT is reasonably low in children undergoing biennial low-dose CT. Harmonization of CT protocols among CF centers should be promoted to comply with the ALARA principle, given that the lifespan of people with CF is increasing.

Statement 15: With a biennial, low-dose CT scheme, the risk related to the cumulative dose (total of nine CTs from 1 to 17 years of age) has been deemed reasonably low.

Statement 16: Further dose reductions could be achieved by introducing a patient-tailored CT imaging follow-up scheme that would stratify the patients with CF according to their risk factors for disease progression, including chronic bacterial infection, pulmonary exacerbation rate, pancreatic insufficiency, nutritional state, age at diagnosis, therapy adherence, and use of CFTR modulators. In patients with CF who are more stable, longer CT scan intervals could allow a reduction of the cumulative dose.

Statement 17: For quality control, the dose reports of the CT examinations should be provided by all centers, to track and manage the radiation dose received by patients with CF.

Statement 18: In people with CF, it is recommended that CT severity scores (e.g., Bhalla, Brody, and Oikonomou) or scores that include the severity of bronchiectasis, bronchial wall thickening, consolidation, and atelectasis be used. The use of software could help reduce the time required for image interpretation and interobserver variability in the assessment.

Statement 19: To reduce radiation exposure, when needed in cooperative children with CF, end-expiratory images should be obtained at a low radiation dose. However, the indication for end-expiratory CT should be carefully considered (i.e., screening or first diagnostic examination) according to the ALARA principle.

Statement 20: The use of appropriate CT scoring systems increases the sensitivity of the examination for tracking changes in symptomatic and asymptomatic early lung disease. Therefore, their use is recommended in order to standardize interpretation of CT data according to CF center expertise and capacity.

Statement 21: In uncooperative children with CF, the recommended CT protocol consists of a free-breathing unenhanced CT without sedation. It should be emphasized that this protocol is very useful for detecting acute pulmonary infection and exacerbation, although it could be of limited utility for assessing some specific initial anatomic abnormalities such as bronchial thickening.

Statement 22: There is no international consensus on CT protocols for patients with CF. Volumetric CT acquisition is recommended, preferably in scanners with 16 or more slices, because of the shorter acquisition time using low tube voltages and currents tailored to the weight or age of the patient, as is the use of reconstruction software that reduces image noise from low-dose CT images.

In conclusion, although CT continues to be a cornerstone in the management and monitoring of CF because of its sensitivity for detecting early disease and tracking progression, its biennial use following the ALARA principle ensures that radiation exposure is kept to a minimum.^5^ The superiority of CT over standard pulmonary function tests in detecting lung disease progression and its ability to provide critical information for modifying patient management, underscores its importance.^6^ Despite advancements in CFTR modulator therapy, the established imaging follow-up protocols should remain unchanged. Although MRI offers a nonradiative alternative with potential benefits, its higher costs, the need for advanced equipment, and other practical limitations restrict its widespread clinical use.
